# Effect of Low Dose of Fumonisins on Pig Health: Immune Status, Intestinal Microbiota and Sensitivity to *Salmonella*

**DOI:** 10.3390/toxins5040841

**Published:** 2013-04-23

**Authors:** Christine Burel, Mael Tanguy, Philippe Guerre, Eric Boilletot, Roland Cariolet, Marilyne Queguiner, Gilbert Postollec, Philippe Pinton, Gilles Salvat, Isabelle P. Oswald, Philippe Fravalo

**Affiliations:** 1 ANSES, Ploufragan-Plouzané Laboratory, BP 53, 22440 Ploufragan, France; E-Mails: mael1.tanguy@laposte.net (M.T.); eric.boilletot@anses.fr (E.B.); roland.cariolet@anses.fr (R.C.); marilyne.queguiner@anses.fr (M.Q.); g.postollec@orange.fr (G.P.); gilles.salvat@anses.fr (G.S.); 2 Ecole Nationale Vétérinaire de Toulouse, Unité de mycotoxicologie, 23 chemin des Capelles, 31076 Toulouse, France; E-Mail: p.guerre@envt.fr; 3 INRA, UMR 1331 ToxAlim, Research Centre in Food Toxicology, 180, chemin de Tournefeuille, BP 93173, 31027 Toulouse Cedex 3, France; E-Mails: philippe.pinton@toulouse.inra.fr (P.P.); isabelle.oswald@toulouse.inra.fr (I.P.O.)

**Keywords:** pig, mycotoxins, fumonisins, growth, immune status, intestinal microbiota, health, *Salmonella*, co-contamination

## Abstract

The objective of this study was to measure the effects of chronic exposure to fumonisins via the ingestion of feed containing naturally contaminated corn in growing pigs infected or not with *Salmonella* spp. This exposure to a moderate dietary concentration of fumonisins (11.8 ppm) was sufficient to induce a biological effect in pigs (Sa/So ratio), but no mortality or pathology was observed over 63 days of exposure. No mortality or related clinical signs, even in cases of inoculation with *Salmonella* (5 × 10^4^ CFU), were observed either. Fumonisins, at these concentrations, did not affect the ability of lymphocytes to proliferate in the presence of mitogens, but after seven days post-inoculation they led to inhibition of the ability of specific *Salmonella* lymphocytes to proliferate following exposure to a specific *Salmonella* antigen. However, the ingestion of fumonisins had no impact on *Salmonella* translocation or seroconversion in inoculated pigs. The inoculation of *Salmonella* did not affect faecal microbiota profiles, but exposure to moderate concentrations of fumonisins transiently affected the digestive microbiota balance. In cases of co-infection with fumonisins and *Salmonella*, the microbiota profiles were rapidly and clearly modified as early as 48 h post-*Salmonella* inoculation. Therefore under these experimental conditions, exposure to an average concentration of fumonisins in naturally contaminated feed had no effect on pig health but did affect the digestive microbiota balance, with *Salmonella* exposure amplifying this phenomenon.

## 1. Introduction

Mycotoxins are toxic secondary metabolites produced by moulds under favourable conditions and which have caused worldwide concern regarding food and feed safety because of their global status and their harmful effects. Pigs are considered to be the farm animals which are the most affected by mycotoxins in general. Horses and pigs are the animals that are the most sensitive to fumonisins. They are affected at levels starting at 0.2 mg FB1/kg bw/day. Fumonisin exposure in pigs is mainly due to ingestion of maize contaminated by *Fusarium verticillioides*. The European Commission has determined maximum levels of fumonisins (FB_1_ + FB_2_) in human food varying from 200 to 2000 ppb [1]. In animal feed, the European Commission currently provides recommendations of maximum levels of fumonisin in raw materials (maize: 60 ppm) and feed (for instance, 5 ppm in feed intended for pigs) [2]. 

As reported by [3] and [4], fumonisin B1 (FB_1_) intoxication of pigs is characterized by functional pulmonary, cardiovascular and hepatic damage. Lethal pulmonary oedema and hydrothorax have been seen in pigs given feed containing more than 12 ppm FB_1_. Recent studies suggest that the intestine is also a target organ for fumonisins [5,6,7,8]. Fumonisins have a molecular structure similar to that of sphinganine (Sa) and sphingosine (So), both of which are cell components of the carbon skeleton of sphingolipids, and which affect the Sa/So ratio. Both [9] and [10] found an increased Sa/So ratio in serum from pigs fed FB_1_ at concentrations of ≥5 ppm. This increase in the ratio is considered to be a dose-dependent early marker of exposure to fumonisins [9,11]. The data found in the literature on the effect of fumonisins on growth performance are inconsistent, but it appears that below a level of 100 ppm FB_1_ in feed, pig performance is most often only slightly affected, or not affected at all, while performance is considerably lower when FB_1_ levels are higher. This reduction generally occurs in pigs presenting intoxication symptoms or tissue lesions [12].

Although lower FB_1_ concentrations do not affect pig performance, it has been demonstrated that intake of a low dosage (6.5–13 ppm FB_1_) of fumonisins increases intestinal colonisation by pathogens such as *Escherichia coli* [13,14] through a decrease in local inflammatory response and a higher permeability of the intestinal epithelium. Similar results were observed with *Salmonella* in the Japanese quail [15], associated with a reduction of the lymphocyte response to infection. Irrespective of the pathological aspects observed in pigs exposed to high concentrations of fumonisins, the chronic intake of low dosages of fumonisins can induce an increase in the proliferation of bacteria such as *Salmonella*, which are major hazards in terms of food safety. Indeed, one of the current priorities in Europe [16] is the control of *Salmonella* *enterica* subspecies *enterica* (*Salmonella* spp) as a zoonotic agent throughout the pig production process. *Salmonella* has several ubiquitous serovars which can contaminate both animal species and humans [17,18]. They are the primary cause of collective food poisoning (CFP) in industrial countries. Pork and pork products were estimated to cause about 15% (range: 7%–20%) of all cases of salmonellosis in industrialized countries (such as the Netherlands, the USA and Germany) [19,20]. Contamination of pig carcasses is linked to asymptomatic carriage of *Salmonella* spp. in the intestinal tract and tonsils of infected pigs. While excretion of *Salmonella* is only intermittent in pigs that are healthy excretory carriers, it represents the main contamination risk for carcasses at the slaughterhouse by exposure to bacteria that are released when contaminated digestive tracts are lacerated. Feeding practices, types of feed and the presence of digestive disorders may influence the persistence of *Salmonella* on pig farms by increasing contamination levels [21]. The role of fumonisins as a factor in disturbances of the intestinal tract remains to be explored.

Indeed, although stability of intestinal microbiota appears to be an important factor for animal health [22], the effect of mycotoxins on this microbiota has been poorly investigated. Bacterial growth of species representative of human intestinal microbiota is not affected by fumonisins [23]. However, feeding pigs with the T-2 toxin resulted in a substantial increase of aerobic bacterial counts in the intestines [24], and surprisingly, in experimental infection with *Salmonella typhimurium* [25], the presence of dietary T-2 toxin led to a reduction in the amount of this pathogen in the caecum contents, and a tendency toward reduced colonisation of the jejunum, ileum, caecum, colon and colon contents was noticed. This effect appeared to be caused by *Salmonella* intoxication by the T-2 toxin. In one of our previous studies [26], we demonstrated that the consumption of feed contaminated with a moderate level of deoxynivalenol (DON) had a slight effect on cultivable bacteria in pig intestines, but in contrast, changes in the composition of intestinal microbiota were observed through Capillary Electrophoresis Single-Stranded Conformation Polymorphism (CE-SSCP) in DON-exposed animals, suggesting that this toxin has an impact on the dynamics of intestinal bacteria communities.

Therefore, the aim of this study was to evaluate the effects of chronic exposure to a moderate level of fumonisins in pigs infected or not by *Salmonella* spp. The impact of exposure to fumonisins was evaluated based on pig growth performance, health status and Sa/So ratio_,_ immune response, bacteriological status, including the dynamics of the total faecal bacterial community, and finally, sensitivity to a *Salmonella* infection.

## 2. Results

### 2.1. Sphinganine/Sphingosine (Sa/So) Ratios in Pig Serum and Tissues

Free sphinganine (Sa) and sphingosine (So) concentrations were measured in serum, kidneys and liver at three dates for serum (2, 9 and 63 days following the start of fumonisin exposure) and at day + 9 and day + 63 for the kidneys and liver (Table 1). 

**Table 1:** Evolution over time of free sphinganine (Sa) and sphingosine (So) concentrations and their ratio (Sa/So) in the groups of pigs exposed to fumonisins (F(+)-S(−) and F(+)-S(+)), compared to the groups not exposed to fumonisins (F(−)-S(−) and F(−)-S(+)), in kidneys, liver and serum.

Days post-fumonisin exposure	Parameter	Groups	Kidneys (nmol/L)	Liver (nmol/L)	Serum (nmol/L)
Day + 2	Sa	F(−)	ND	ND	15.36 ± 1.26
		F(+)	ND	ND	16.56 ± 1.30
	So	F(−)	ND	ND	76.67 ± 6.24
		F(+)	ND	ND	77.07 ± 6.22
	Sa/So	F(−)	ND	ND	0.20 ± 0.01
		F(+)	ND	ND	0.22 ± 0.01
Day + 9	Sa	F(−)	0.32 ± 0.02 ^a^	0.40 ± 0.03 ^a^	12.65 ± 1.77
		F(+)	0.46 ± 0.03 ^b^	0.96 ± 0.11 ^b^	13.15 ± 0.57
	So	F(−)	5.86 ± 0.39	3.32 ± 0.24 ^a^	76.45 ± 10.55
		F(+)	7.18 ± 0.68	5.30 ± 0.56 ^b^	54.91 ± 2.76
	Sa/So	F(−)	0.05 ± 0.00 ^a^	0.12 ± 0.01 ^a^	0.17 ± 0.01 ^a^
		F(+)	0.07 ± 0.00 ^b^	0.18 ± 0.00 ^b^	0.24 ± 0.01 ^b^
Day + 63	Sa	F(−)	0.33 ± 0.03 ^a^	0.31 ± 0.03	7.14 ± 0.52 ^a^
		F(+)	0.72 ± 0.10 ^b^	0.47 ± 0.07	18.10 ± 1.75 ^b^
	So	F(−)	6.88 ± 0.71 ^a^	2.94 ± 0.36	54.34 ± 3.46
		F(+)	9.28 ± 0.81 ^b^	3.05 ± 0.64	55.78 ± 4.90
	Sa/So	F(−)	0.05 ± 0.00 ^a^	0.11 ± 0.00 ^a^	0.13 ± 0.00 ^a^
		F(+)	0.08 ± 0.00 ^b^	0.17 ± 0.01 ^b^	0.33 ± 0.02 ^b^

Data (means ± SEM; *n* = 8) have been statistically analyzed for each date using the *t*-test. For each parameter (Sa, So and Sa/So) and each date, the presence of superscripted letters indicates a significant difference between the two values (*p* < 0.05). day + 2, day + 9 and day + 63 after the beginning of fumonisin exposure correspond to D − 5, D + 2 and D + 56 post-inoculation with *Salmonella*, respectively; ND: Not Determined.

No significant differences were observed at day + 2 in the serum of the F(+) and (F−) animals. However significant differences appeared as of day + 9 where the Sa/So ratio slightly but significantly increased regardless of the tissue observed. Fumonisins at a level of 11.8 ppm were therefore responsible for a statistically significant increase in the Sa/So ratio starting nine days after the beginning of exposure.

### 2.2. Growth Performance and Health Status

No mortality or disease (clinical observations) caused by mycotoxicosis was observed during the 63 days of exposure of our pigs to a diet containing 11.8 ppm of fumonisins. Nor were any clinical signs associated with *Salmonella* inoculation found, as expected. The body temperature of the pigs was 39.3 ± 0.3 °C (min 38.5 °C, max 40.9 °C). In addition, neither the overall growth performance of our pigs, nor their total feed intake, was affected by any of the experimental treatments. However, a transient decrease in feed intake was observed at D − 7 in all pig groups during the transition period between administration of initial piglet feed and the switch to experimental follow-on pig feed. With feed consumption and pig weight taken into account, the pigs received, on average, 0.46 mg/kg/day of FB1 and 0.17 mg/kg/day of FB2 (=0.63 mg FB1 + FB2/kg/day) during this study.

### 2.3. Serologic Status

The qualitative serology analysis reflected changes over time in the number of specific *Salmonella* seroconversions in inoculated pigs S(+). The first seroconversion occurred two weeks post-inoculation. The number of seropositive pigs seemed to be higher in the F(−)S(+) group than in the F(+)S(+) group as of D + 21 (Table 2). This difference is significant at D + 28 (*p* = 0.04). Concerning the seropositive pigs, the intensity of seroconversion was not significantly different (mean *X* of the serums varying between 0.7 and 1.7, data not shown) in the two groups of pigs (*X* > 0.4). Exposure to 11.8 ppm of fumonisins had no effect on the intensity of seroconversion.

**Table 2:** Changes over time in the number of specific *Salmonella* seroconversions in inoculated pigs (eight per group).

Groups Time	D + 2	D + 7	D + 14	D + 21	D + 28	D + 35	D + 42	D + 49
F(−)S(+)	0/8	0/8	1/8	7/8	7/8^b^	7/8	7/8	7/8
F(+)S(+)	0/8	0/8	2/8	4/8	2/8^a^	3/8	3/8	4/8

*Salmonella* inoculation performed at D0. The difference between proportions (*n* = 8) have been statistically analyzed for each date using the Fisher’s Exact Test. For each date, the presence of superscripted letters indicates a significant difference between the two values (*p* < 0.05).

### 2.4. Immune Status

#### 2.4.1. Non-Specific Cellular Immune Response

A dietary concentration of 11.8 ppm of fumonisins did not affect the capacity of lymphocytes to proliferate in the presence of the two mitotic agents tested (Concanavaline A and PMA plus Ionomycin) in the F(+) pigs (data not shown). It should also be noted that no deleterious effects due to *Salmonella* inoculation were observed.

#### 2.4.2. Specific Immune Response

**Figure 1:**
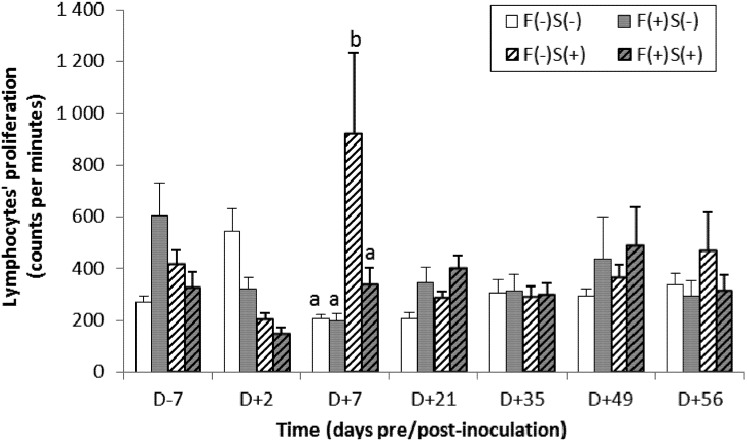
Changes over time in the proliferation capacity of lymphocytes in the presence of a *Salmonella*-specific antigen in pigs fed a diet containing fumonisins F(+) or not F(−) and inoculated with *Salmonella* S(+) or not S(−). Data (means ± SEM, *n* = 12 then *n* = 8 after D + 2) were statistically analyzed for each date using variance analysis (ANOVA) followed by the Tuckey test. The small letters (“a” and “b”) indicate a significant difference (*p* < 0.05).

A specific cellular response to *Salmonella* was observed in the F(−)S(+) pig group at D + 7. In fact, lymphocyte proliferation was significantly higher following exposure to *Salmonella* lipopolysaccharide (LPS) (Figure 1). However, this response was heterogeneous and transient, since only some of the animals were affected and this parameter returned to a basal level the following week.

### 2.5. Bacteriological Status of the Animals

#### 2.5.1. Changes over Time in the Percentage of Excretory Pigs and in the Intensity of Excretion

The faeces of the two groups of pigs not inoculated with *Salmonella*, F(−)S(−) and F(+)S(−), were *Salmonella typhimurium*-free throughout the experiment (data not shown). In the inoculated pigs, the number of excretory pigs was initially higher (at D + 2) in the F(+)S(+) group than in the F(−)S(+) group, and close to 100% of all the animals (Table 3). Two weeks later, only 50% of the pigs were still excretory and this proportion remained stable until D + 49. The situation was reversed in the F(−)S(+) group in which inoculation delayed excretion and after two weeks all the pigs were excretory. This situation remained stable until D + 49.

All faeces of the excretory pigs presented *Salmonella* concentrations close to 5 Log MPN/g up to two weeks post-inoculation. The intensity of excretion then decreased, first in the F(+)S(+) group (from D + 14), followed by the F(−)S(+) group (from D + 21) (Table 3). However, no statistical differences (*p* > 0.05) were detected between the two groups of pigs in terms of faecal *Salmonella* concentration, and therefore no significant impact of FB_1_. 

**Table 3:** Changes over time in the number of pigs excreting *Salmonella typhimurium* and in *Salmonella* concentrations in the faeces of positive animals.

		Time (days pre-/post-inoculation)								
		D − 7	D + 2	D + 7	D + 14	D + 21	D + 28	D + 35	D + 42	D + 49
Excretory pigs ^1^	F(−)S(+)	0	8/12	7/8	8/8	7/8	7/8	7/8	7/8	7/8
	F(+)S(+)	0	11/12	5/8	4/8	4/8	4/8	4/8	4/8	4/8
*Salmonella* concentration ^2^	F(−)S(+)	-	2.7 ± 0.7	2.9 ± 0.3	2.7 ± 0.2	1.2 ± 0.3	1.1 ± 0.5	0.4 ± 0.5	0.7 ± 0.5	1.1 ± 0.6
	F(+)S(+)	-	2.7 ± 0.2	2.9 ± 0.3	1.6 ± 0.6	1.2 ± 0.5	1.1 ± 0.5	0.8 ± 0.4	1.4 ± 0.8	1.0 ± 0.5

^1^ Number of pigs presenting *Salmonella*-contaminated faeces (out of the total number of observed pigs); ^2^ Level of *Salmonella* contamination (log: Log MPN/g) in the positive animals. Data (means ± SEM) were statistically analyzed for each date using the *t*-test (*p* < 0.05).

#### 2.5.2. Research and Counting of *Salmonella typhimurium* in Intestinal Contents and Tissues

The bacterial investigations of intestinal contents and of certain tissues confirmed that the two groups of pigs not inoculated with *Salmonella*, F(−)S(−) and F(+)S(−), remained *Salmonella typhimurium*-free throughout the experiment (data not shown). 

**Table 4:** Detection of *Salmonella typhimurium* in intestinal contents and organs of the pigs from groups F(−)S(+) and F(+)S(+) 2 days and 56 days post-inoculation.

	D + 2 (*n* = 4)				D + 56 (*n* = 8)			
	F(−)S(+)		F(+)S(+)		F(−)S(+)		F(+)S(+)	
	Nb ^1^	Conc. ^2^	Nb ^1^	Conc. ^2^	Nb ^1^	Conc. ^2^	Nb ^1^	Conc. ^2^
Liver, spleen, muscle	0/4	-	0/4	-	0/8	-	0/8	-
Mesenteric nodes	2/4	1.3 ± 0.5	3/4	1.6 ± 0.4	0/8	-	2/8	<0.1
Ileum content	2/4	1.7 ± 0.6	2/4	2.4 ± 0.6	3/8	2.1 ± 0.6	3/8	2.7 ± 0.6
Colon content	3/4	0.5 ± 0.3	3/4	2.0 ± 1.4	6/8	1.6 ± 0.4	3/8	2.4 ± 0.4
Caecum content	3/4	<0.1	3/4	2.1 ± 1.9	7/8	2.1 ± 0.4	3/8	2.6 ± 0.7

^1^ Number of pigs presenting *Salmonella-*contaminated intestinal contents or tissues (out of the total number of observed pigs); ^2^ Level of *Salmonella* contamination (log: Log MPN/g) in the positive animals. Data (means for positive animals ± SEM) were statistically analyzed for each date and each type of sample using the *t*-test (*p* < 0.05).

In the S(+) pigs, inoculation led to contamination of the mesenteric nodes, where *Salmonella* was detected in more than 50% of the pigs at D + 2 (Table 4). They were still contaminated at D + 56, but only in the F(+)S(+) pigs and to a lesser extent. However, the differences were not significant in the F(−)S(+) and F(+)S(+) groups, *i.e.*, no fumonisin effect was detected with regard to mesenteric node contamination. However, *Salmonella* was not found, either at D + 2 or at D + 56, in the muscle, liver or spleen samples. Concerning the intestinal contents studied (ileum, colon and caecum), the proportion of *Salmonella*-positive samples was high (over 50%; *n* = 4) at D + 2. However dietary contamination with 11.8 ppm of fumonisins had no significant impact on the contamination level two days post-inoculation, and only showed a tendency (*p* = 0.09), despite a 1.4 Log difference between the two groups of pigs, as demonstrated by the quantitative analyses (Table 4). The proportion of positive animals at D + 56 (*n* = 8) seemed to be inferior to that measured at D + 2, but this difference remained small. In addition, levels of excretion were similar and no impact from dietary fumonisins was detected.

#### 2.5.3. Study of the Translocation of *Salmonella* into Organs

Immunodetection of *Salmonella* was performed on transversal slides of the mucosa of the ileo-caecal junction at the level of the Peyer’s patches. Bacteria were observed only in the most highly-excretory pigs, without any differences between the F(−)S(+) and F(+)S(+) groups with regard to the number of bacteria per field or to their localisation, strictly limited to the apical area of the pigs’ mucosa. Dietary exposure to 11.8 ppm of fumonisins made no difference with regard to the mucosal localisation of *Salmonella*. No increase in translocation related to fumonisin intake was observed.

### 2.6. The Intestinal Microbiota of the Animals

#### 2.6.1. Mesophylic Aerobic Counts

Counting of aerobic mesophylic bacteria (AMB) was conducted on the faeces and intestinal contents of the pigs throughout the experiment in order to screen for a potential imbalance. No imbalances were highlighted during the study regardless of the treatment: the number of AMB was estimated at between 10^7^ and 10^8^ CFU per gram of faeces throughout the experiment. A count was also performed on the ileal, caecal and colonic contents of pigs at D + 2 and D + 49 post-inoculation. No significant differences between treatments, either for pigs slaughtered at D + 2 or for pigs slaughtered at the end of the trial (D + 49), were observed: the number of AMB was estimated at between 10^7^ and 10^8^ CFU per gram of material in the ileum and colon and at 10^7^ CFU per g in the caecum, regardless of the date of sampling.

#### 2.6.2. Faecal Microbiota SSCP-Profiles

Prior to analysis, we observed that a combination of the DNA from four pigs generated the most representative profile of the group to which they belonged (data not shown). We therefore prepared, for D − 6, D + 2, D + 8, D + 22 and D + 49, mixtures of four faecal DNA samples randomly selected for each experimental treatment (F(−)S(−), F(+)S(−), F(−)S(+) and F(+)S(+)).

**Figure 2:**
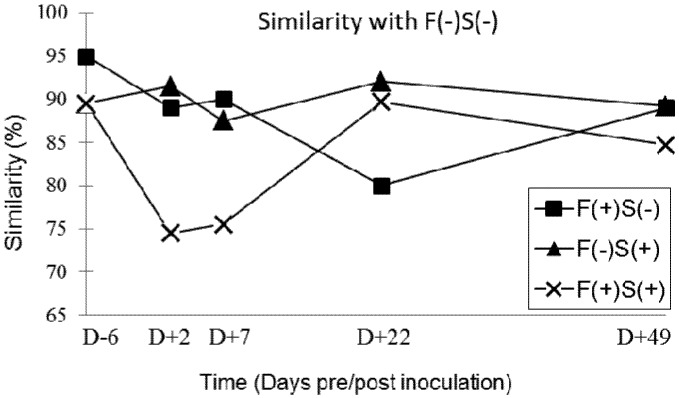
Changes over time in the similarity between the profile of the faecal microbiota of the control group F(−)S(−) and those of the other groups of pigs: F(+)S(−), F(−)S(+) and F(+)S(+).

Figure 2 shows the changes over time in the similarity between the profile obtained from the faecal microbiota of the control group F(−)S(−) and those of the F(+)S(−), F(−)S(+) and F(+)S(+) groups. A transient but marked decrease in similarity between the profile of the control group F(−)S(−) and that of the F(+)S(+) group was observed during the study. Indeed, the similarity between these two groups decreased from 89.2% to 74.2% between D − 6 and D + 2, stabilized between D + 2 and D + 7 and then increased by 75.8% to 84.9% between D + 7 and D + 49. A later transient decrease of the similarity between the control group and the F(+)-S(−) group was also observed: a decrease from 90.2% to 80.1% between D + 7 and D + 22, followed by the return to a “normal” level (88.9%) between D + 22 and D + 49. 

## 3. Discussion

This study examines the impact of chronic ingestion of low doses of fumonisins in pigs subjected or not to asymptomatic carriage of *Salmonella typhimurium*. This study provides data that help to determine the public health risk of the association of a fumonisin-contaminated diet with *Salmonella* carriage. However, it must be kept in mind that this study was performed with specific pathogen-free (SPF) pigs obtained as described in [27]. These pigs express better growth performance and have much better health status than conventional pigs raised in standard pig farms.

### 3.1. Intoxication of Pigs by Fumonisins

The feed used in this study contained corn naturally contaminated with fumonisins at a level of 100 ppm of FB_1_ and 30 ppm of FB_2_, levels which are occasionally observed in France. The feed itself contained 8.6 ppm FB_1_ and 3.2 ppm FB_2_, a toxin level which induced a slight increase in the Sa/So ratio in our pigs, confirming their intoxication by fumonisins, although its intensity seemed to be quite low. An increase in this ratio indicates that an animal has been exposed to fumonisins, and it is particularly appropriate in the context of natural exposure to the toxin [9,28].

As part of our study and under our experimental conditions, feed contaminated with 11.8 ppm of fumonisins had no effect whatsoever either on animal performance (feed consumption, growth, feed efficiency) or on the health of the animals as determined by clinical examination (clinical signs, body temperature, necropsies). These results are consistent with those of previous studies performed on conventional piglets fed diets containing 1 to 10 ppm FB_1_ for 4 to 8 weeks [10,29,30]. These studies did not show any deleterious effects of fumonisins on either the performance or health of piglets. Another study, however, reported erratic growth in conventional pigs at a concentration of 0.1 ppm FB_1_ in the diet and reduced growth at a concentration of 1 ppm of FB_1_ [31]. These differences could be caused by differences in experimental conditions, animal health status, interactions with other contaminants, *etc*.

The autopsies performed in our study (at D + 2 and D + 49) failed to demonstrate any pulmonary œdema or specific lesions at macroscopic scale. Previous studies tend to confirm this result. Thus, [29,32] showed no clinical effects, including pulmonary œdema, during contamination with 10 ppm of FB_1_ in the diet of weanling pigs. However, they showed a decrease in body weight gain and food consumption, and the presence of pulmonary oedema at a dietary concentration of 30 ppm of FB_1_ in all animals that received the contaminated feed during a period of 28 days. However our results are in conflict with those of [30] and [10], which showed at autopsy the presence of pulmonary oedema in 2 out of 5, and 3 out of 4, piglets fed with a diet contaminated with 5 ppm and 10 ppm FB_1_, respectively. Nevertheless, in pigs fed a diet contaminated with 6 ppm FB1 + FB2, microscopic pulmonary lesions were observed with no macroscopic signs of pulmonary lesion [33]. In addition, the conventional pigs used in these studies [10,30,33] may have had a lower health status (*i.e*., prior lesions of the digestive and respiratory systems for instance) than our SPF animals.

### 3.2. Impact of FB_1_ on the Asymptomatic Carriage of *Salmonella*

*Salmonella* in pig production is a worldwide concern for food hygiene, since asymptomatic carriage of *Salmonella* in the gastrointestinal tracts of pigs, followed by the contamination of carcasses during the evisceration process at the slaughterhouse, represents a significant risk of foodborne disease. On the farm, this carriage does not lead to pathology. The conditions employed in our study reproduced asymptomatic carriage of *Salmonella*. It requires that within 15 days post-inoculation pathogen concentrations be close to 5 log CFU/g, and faecal contents should later contain approximately 100 *Salmonella* per gram of faeces [34]. This was the case in our study.

Our results showed that FB_1_ had no impact on the number of excreting pigs, exposed or not to fumonisins, while almost all of them were excretory at least for a short period. In addition, FB_1_ had no impact on the intensity of excretion. It would however appear that intake of FB1 led to more rapid excretion (two days post-inoculation), and a quicker decrease in intensity (two weeks after inoculation) as compared to pigs not fed with an FB_1_ diet. The quantitative assessments performed on intestinal contents showed a 1.4 log difference between the two groups of infected pigs; nevertheless this difference did not reach levels of statistical significance and remained a trend. Ingestion of our contaminated feed therefore did not strongly affect either *Salmonella* carriage or shedding. Moreover, no translocation at the Peyer’s patches was associated with its consumption.

All the parameters measured lead us to conclude that under our experimental conditions, a diet containing 11.8 ppm of fumonisins has no effect on the asymptomatic carriage of *Salmonella enterica*. This result is in contradiction with [5,6,7,8] which predicted that chronic ingestion of low doses of mycotoxins, including FB1, would damage the intestine, and thus could predispose pigs to infections by enteric pathogens. Indeed, these authors demonstrated that ingestion of FB1 (6 ppm) induced morphological and histological changes in the intestine, with atrophy and fusion of the villi, decreased villi height and cell proliferation in the jejunum, and reduced numbers of goblet cells and lymphocytes. In addition, [15] showed in quail that FB_1_ could increase the severity of infection by *Salmonella* Gallinarum, a typhoid serovar for birds. However, the dietary concentration of FB_1_ was very high (150 ppm) in the quails’ feed, compared to the concentration used in our study. This study also showed that the increased susceptibility of quail to *Salmonella* Gallinarum infection could be attributed to suppression of the immune response, as evidenced by a decrease in the number of lymphocytes in the presence of fumonisins.

### 3.3. The Impact of FB1 on Immune Status and Bacterial Infection

In our study, seroconversion to *Salmonella* appeared two weeks post-inoculation in pigs inoculated with *Salmonella*, and surprisingly the number of seropositive animals was lower in the group exposed to fumonisins than in the unexposed group. However, exposure to fumonisins did not affect the intensity of this seroconversion in the seropositive animals. The effect of FB_1_ on the number of seroconverted pigs could be caused by the toxin’s impact on the immune capacity of certain pigs.

Mycotoxins can alter immune response [3,35] and consequently increase the sensitivity of animals, including pigs, to infectious pathogens, as demonstrated by several authors using experimental infection. This is the case with intestinal disorders caused by *Escherichia coli* [13,14], *Pasteurella multocida* [36] and respiratory disorders in pigs caused by *P. multocida* and *Bordetella bronchiseptica* [37] together. In the case of intestinal pathogens, the two studies (see Table 5) demonstrated that FB1 can intensify the infection: increased colonisation of the small and large intestines with inoculated *E. coli*, associated with lower induction of antigen-specific immune response in the study by [13], and longer shedding associated with reduction of the mucosal immune response in the study by Devriendt [14].

**Table 5:** Comparison of the experimental designs of three studies (including ours) and the results obtained concerning the potential predisposing effect of fumonisins to intestinal pathogens in pigs.

	Oswald *et al*. (2003)	Devriendt *et al*. (2009)	Our study
Age of pigs at FB1 exposure	3 weeks	4 weeks	7 weeks
Status of pigs	Conventional	Conventional	SPF
Estimated FB1 dietary concentration	6.5 ppm FB1	13 ppm FB11.9 ppm FB22.2 ppm FB3	8.6 ppm FB13.2 ppm FB2
FB1 presentation	Crude extract	Crude extract	Maize naturally contaminated
FB1 distribution	Gavage	Gavage	In feed
Age of pigs at inoculation	4 weeks	6 weeks	8 weeks
Pathogens inoculated	*E. coli* (ExPEC strain) 1 × 10^9^ CFU	*E. coli* (F4^+^ ETEC strain) 10^10^ CFU	*S. typhimurium* 5 × 10^4^ CFU
FB1 as predisposing factor to disease	Yes	Yes	Questionable

CFU: colony forming unit.

In our study, despite the high invasiveness of *Salmonella*, there was no phenomenon of translocation associated with exposure to fumonisins, and no effect of the fumonisins on the ability of lymphocytes to proliferate was observed. These results contradict those of [13] which showed that oral administration of purified FB_1_ resulted in increased invasiveness of pathogenic *Escherichia coli* through the intestinal barrier. This effect could be attributed to the negative impact of FB1 on the intestinal barrier. A study has shown that FB1 inhibits proliferation of the porcine intestinal epithelial cell line and has a deleterious effect on the capacity of these cells to form a mono-layer [38]. In fact, FB1 alters the intestinal barrier function by influencing sphingolipid metabolism, as demonstrated by an increase in the amount of free sphingoid bases, a depletion of glycolipids in the plasma membrane and an increase in trans-epithelial flux [38,39]. Obviously, the impact of FB1 on sphingolipid metabolism observed in our study (change in the Sa/So ratio) was not sufficient to affect the integrity of the intestinal epithelium. In addition, under our experimental conditions, fumonisins had no effect on the nonspecific cellular response to infection, in contrast to the observations made by [13] and [40], *i.e.*, decreased levels of mRNA coding for inflammatory cytokine IL-8 in the enterocytes of the ileum and other components of the innate immune response that are first line defence mechanisms [5]. Of course, the two studies were designed quite differently (Table 5): (i) *Salmonella* vs. *E. coli*; (ii) crude extract of FB1 *vs*. naturally contaminated feed (although the final dosages were quite similar); (iii) younger pigs in [13] *vs*. older pigs in our study, and finally (iv) our pigs were SPF (specific pathogen free) while those of [13] were conventional.

However, in our study we observed an inhibition of the specific parameters of cellular response to *Salmonella* in pigs exposed to fumonisins: an impaired ability of *Salmonella*-specific lymphocytes to proliferate in the presence of a selective mitotic agent. However, this response was transient and heterogeneous. However, a significant increase in lymphocyte growth was also observed in some animals infected with *Salmonella* but not exposed to fumonisins. In this context, the effect of exposure to fumonisins on lymphocyte proliferation remains to be confirmed. However, if this effect of FB_1_ on adaptative immunity is assessed, it would be in agreement with those of [41] and [14] (Table 5). Indeed, [41] showed that FB1 reduces the efficiency of intramuscular vaccination, and [14] observed, using their infectious model with *E. coli* F4^+^, that FB1 could reduce induction of an antigen-specific intestinal immune response: lower numbers of antigen-specific IgM antibody-secreting cells were detected in the jejunal Peyer’s patches of FB1-exposed piglets. Their result suggests that FB1 could interfere with the induction phase of the immune response, especially since the overall mucosal IgA immune response was significantly reduced in FB1-exposed piglets. Further analyses to elucidate the mechanisms behind these observations revealed reduced intestinal expression of IL-12p40, impaired function of intestinal antigen-presenting cells, with decreased up-regulation of Major Histocompatibility Complex Class II molecule (MHC-II) and reduced T-cell stimulatory capacity upon stimulation. Another study [5,6,7,8] demonstrated that the expression levels of several cytokines (TNF-α, IL-1β, IFN-γ, IL-6 and IL-10) were significantly up-regulated in the ileum or the jejunum of pigs fed a diet containing 6 ppm FB_1_. In addition, the ingestion of contaminated diets reduced expression of the adherent junction protein E-cadherin and the tight junction protein occludin in the intestine. Taken together, these results indicate an FB1-mediated reduction of in vivo APC maturation, which could explain the longer F4 + ETEC excretion and the lower F4-specific immune response to oral F4 immunisation in FB1-exposed animals [14].

### 3.4. Faecal Microbiota Profiles in Groups of Pigs Exposed to Fumonisin and/or *Salmonella*

In our study, we created DNA mixtures from the faeces of four individuals in order to represent the profile of each group and to study the effect of exposure to fumonisins and/or *Salmonella* on faecal microbiota.

Our results showed that chronic exposure to 11.8 ppm of fumonisins transiently affects the balance of the digestive microbiota during the first four weeks of exposure. The imbalance then diminishes over the following four weeks of feeding and at the end of the trial all profiles had once again become very similar. However, there was no significant effect of the inoculation of 5 × 10^4^ CFU of *Salmonella* and of asymptomatic carriage of *Salmonella* on the balance of the intestinal microbiota of our pigs. However, in cases of co-contamination, the balance of the digestive microbiota was also affected after nine days of exposure to dietary fumonisins and after two days of *Salmonella* infection. This phenomenon was also transient, but faster and more intense than what was observed with exposure to fumonisins alone. Thus, exposure to 11.8 ppm of fumonisins along with colonisation of the digestive tract by *Salmonella* was likely to favour a digestive imbalance. A return to the initial microbiota balance was then observed from 22 days post-inoculation and remained constant up the end of the experiment. The single inoculation with 5 × 10^4^ CFU of *Salmonella* did not induce any changes in the digestive balance when measured by the SSCP method, but this type of infection might be able to amplify the destabilizing effect on microbiota of feed contaminated with 11.8 ppm of fumonisins. Further studies would be necessary to confirm this hypothesis.

The mechanism of action of fumonisins on eukaryotic cells is well documented but the effect of this toxin on intestinal microbiota is largely unknown. In this study, we demonstrated for the first time that fumonisins modify the intestinal microbiota of animals, although further analyses are needed to better identify the observed changes and more specifically to identify the bacteria involved. However, these results are in agreement with those by [26] which also used the CE-SSCP method in SPF pigs, and demonstrated changes in the intestinal microflora of young pigs receiving a diet naturally contaminated with DON (2.8 ppm). The intestinal microbiota is very important because there is a strong relationship between the host and its intestinal microbiota, especially through the immune response, resistance to pathogenic pathogen colonisation, and via the metabolic products of the fermentation processes. Thus an impaired balance of the intestinal flora could have many adverse effects on the health of the host [42]. This may be the reason that we observed an interaction between fumonisins, *Salmonella* infection and immune response, although the extent of the observed changes remained low. It would be interesting to test the impact of fumonisins in the microbiota of conventional pigs in which both the microbiota and health status are very different compared to what is observed in SPF animals. In addition, as weaned piglets are very susceptible to developing intestinal disorders during this critical period of dietary transition, it would be of great interest to evaluate the impact of fumonisins on the microbiota of these very young animals.

## 4. Experimental Section

### 4.1. Animals and Housing

**Figure 3:**
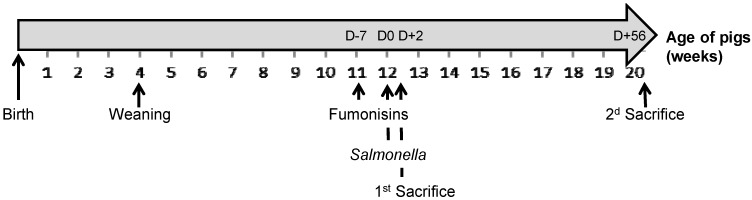
Diagram representing the main information in the experimental design according to the age of the pigs: weaning of all piglets, beginning of distribution of fumonisin-contaminated feed to the F(+) pigs at D − 7, *Salmonella* inoculation of the S(+) pigs at D0, first and second pig sacrifice dates at D + 2 and D + 56, respectively.

This animal experiment was carried out in strict accordance with the Guidelines of the National Institutes of Health Guide and the French Ministry of Agriculture for the care and use of laboratory animals. The study used 48 four-week-old specific pathogen-free (SPF) Large-White piglets born at the ANSES experimental farm (Ploufragan, France). The day of weaning (at 28 days of age), animals were individually identified and divided into four experimental groups. The piglets came from six different litters and included 1/3 females and 2/3 castrated males, with an initial mean weight of 41.6 kg at D − 7 (see Figure 3). Animals were allocated to the different groups taking into account sex and the life body weight of the piglets. The study was carried out in the experimental confined facilities for pigs of ANSES. Each group, divided into two pens of six animals, was housed in a separate block of the housing unit with free access to feed and water. Extensive swab samplings were taken from the soil of each pen at D − 27 and D − 12, before inoculation, in order to screen for *Salmonella* and to check the housing system and the SPF status of the animals. 

### 4.2. Feed

Pigs were fed *ad libitum* and the feed distributed manually once a day to the different groups was weighed. Feed refusals were weighed and discarded twice a week. The pelleted diets were prepared locally. They were formulated according to the energy and nutrient requirements of growing pigs (follow-on pig feed). Two different batches of maize, provided by ARVALIS-Institut du Végétal (France), were used. One control batch was free of fumonisins contamination and the second batch was naturally contaminated with about 130 ppm of fumonisins. The F(+) diet contained 15% of the contaminated maize, the objective being to obtain a F + diet containing 20 ppm of FB_1_. However, the final concentrations in the contaminated feed were lower: 8.6 ppm FB1 and 3.2 ppm FB2 (11.8 ppm FB1 + FB2) (Table 6). 

Mycotoxins were analysed using Liquid Chromatography coupled with tandem Mass Spectrometry (LC-MS/MS) techniques and their detection limit was established between 0.5 and 50 ppb feed according to the type of mycotoxin. Measurements in maize samples and in feed samples were performed by two different laboratories, which might explain the difference between the expected and the final fumonisin concentrations. Results of the analyses confirmed that the experimental diets were contaminated with fumonisins. Only very low amounts of deoxynivalenol (DON) were measured in the F(+) diet, and also in the control diet F(−) (Table 6).

**Table 6:** Ingredients, nutrient composition and mycotoxin contamination of the experimental diets.

Diets	Control F(−)	Contaminated F(+)
Ingredients (%)		
Uncontaminated maize	20.4	5.4
Contaminated maize	0	15
Barley	45	45
Soya meal 48	16	16
Dehydrated alfalfa	10	10
Oats	4	4
Calcium phosphorus	2	2
Vitamins, minerals, oligo-elements	2.7	2.7
Calculated composition ^1^		
Dry matter (g/kg)	874	
Whole Cellulose (g/kg)	66	
Fat (g/kg)	24	
Starch (g/kg)	385	
Net energy (MJ/kg)	8.63	
Gross Protein (g/kg)	157	
Ash (g/kg)	68	
Measured mycotoxin content (ppb) ^2^		
Fumonisin B1 (FB_1_)	ND (<10)	8616
Fumonisin B2 (FB_2_)	ND (<50)	3205
FB_1_ + FB_2_	ND (<50)	11,821
Aflatoxin B_1_	<0.1	<0.1–0.24
Ochratoxin ^3^	ND (<0.5)	ND (<0.5)
Zearalenone	ND (<40)	ND (<40)
Trichothecenes type A ^4^		
Monoacetoxyscirpenol	ND (<40)	ND (<40)
Diacetoxyscirpenol	ND (<40)	ND (<40)
T-2 toxin	ND (<50)	ND (<50)
T-2 triol	ND (<40)	ND (<40)
T-2 tetraol	ND (<40)	ND (<40)
HT-2 toxin	ND (<40)	ND (<40)
Trichothecenes B ^4^		
Deoxynivalenol	112–131	64–114
Neosolaniol	ND (<40)	ND (<40)
3 acetyl-deoxynivalenol	ND (<40)	ND (<40)
15 acetyl-deoxynivalenol	ND (<40)	ND (<40)
Fusarenon X	ND (<40)	ND (<40)
Nivalenol	ND (<50)	ND (<50)

^1^ The composition of the diets was calculated based on the composition of the feedstuffs and their respective percentage of incorporation into the feed; ^2^ Mycotoxin content was measured by a private laboratory (Laboratoire de Développement et d’Analyses des Côtes d’Armor, Ploufragan, France); ^3^ Method NF EN 12955; ^4^ Method 7D MA 6–24; ND: not detectable.

### 4.3. Experimental Design

The two-by-two factorial experimental design aimed to test the cross effect of fumonisin intake F(+) or not F(−) and of *Salmonella* inoculation S(+) or not S(−). Four experimental treatments were tested: F(−)S(−), F(−)S(+), F(+)S(−) and F(+)S(+). The 12 pigs from each F(+)S(−) and F(+)S(+) group were fed the contaminated diet from 7 days before inoculation (D − 7; at 11 weeks old) and up to the end of the experiment (D + 56), while the 12 pigs from each F(−)S(−) and F(−)S(+) group were fed the uncontaminated diet during the whole duration of the experiment. The 24 pigs from the F(−)S(+) and F(+)S(+) groups were individually inoculated at D0 (at 12 weeks old) through a single oral administration of 5 × 10^4^ *Salmonella enterica* Typhimurium in order to provoke asymptomatic carriage and excretion, while placebo inoculation was performed on the 24 pigs from the F(−)S(−) and F(+)S(−) groups the same day (according to a previously developed model [34]). The full duration of the experiment was 9 weeks following the start of fumonisin exposure. Four pigs per treatment (*i.e.*, 2 pigs/pen) were sacrificed 48 hours post-inoculation (D + 2). All the remaining pigs were then sacrificed at the end of the experiment (D + 56) (Figure 3).

### 4.4. Animal Growth Performance and Sample Collection

Pigs were weighed weekly. The daily weight gain was calculated per animal and feed intake was measured per pen and measured weekly. The feed conversion ratio was calculated by week and per pen based on consumption.

Faecal samples were individually collected from all pigs at D + 2 and then every week starting on the inoculation date (D0). Samples were immediately divided into two fractions: one fresh fraction was used to count intestinal bacterial populations by cultural methods (see bacteriological counts), while the other was stored at −80 °C and then used to perform molecular analysis by Capillary Single-Stranded Conformation Polymorphism (see CE-SSCP). 

Blood samples were taken from pigs once a week at the beginning of the trial, then every two weeks. Blood was divided into two fractions. One fraction was immediately centrifuged to obtain serum stored at −20 °C until analysis. Serum samples were used to measure the sphinganine/sphingosine ratio (Sa/So) and to measure the duration and intensity of the specific sero-conversion against *Salmonella.* The second blood fraction was directly used to evaluate the blood immune status of the pigs by quantification of specific *Salmonella* lymphocytes proliferation.

Pigs were autopsied 2 days and 56 days post-inoculation and diaphragm muscle, mesenteric lymph nodes (MLN), the liver, spleen, and ileo-caecal junction, as well as the jejunum, caecum and descending colon contents were sampled. The liver, spleen and ileo-caecal junction were immediately quick-frozen in liquid nitrogen. The pigs’ exposure levels to fumonisins was evaluated by measuring the Sa/So ratio in the kidney, liver and in serum. The localisation, colonisation, accumulation and tissue distribution of *Salmonella* in pigs was evaluated in the liver, spleen, muscle, MLN, and ileum, descending colon and caecum contents. A *Salmonella* immuno-histochemistry analysis was performed at the level of the ileo-caecal junction. Finally, a numeration of the mesophilic aerobic microbiota was performed on the jejunum, caecum and descending colon contents. 

### 4.5. Evaluation of Fumonisin Exposure: The Sa/So Ratio

The free sphinganine (Sa) and sphingosine (So) concentrations were determined by HPLC following the method described by [9]. 5 µL of C_20_ sphinganine internal standard solution (1 µM, obtained from Biovalley, Marne-La-Vallée, France) was briefly added to 100 µL of serum or tissue homogenate. Lipids were extracted using a chloroform/methanol (1:2 *v*/*v*) solution. Lipids were then hydrolyzed to release Sa and So by addition of NH_4_OH (2 N) and heated at 37 °C for 1 h. After cooling down to ambient temperature, 1 mL of CHCl_3_ and 2 mL of alkaline water were added and samples were mixed and centrifuged 10 min at 100*g*. The aqueous phases were thrown out and the CHCl_3_ phases were washed twice with alkaline water. Samples were then dried and 20 µL of methanol was added. The obtained extracts were derivatised with *o*-phthaldialdehyde overnight at 4 °C. Concentrations in Sa, So and C_20_ Sa were determined by HPLC using an ICS M2200 solvent connected to a programmable FD-500 fluorescence detector, an analytical Radial-pak cartridge combined with the Nova-pak C_18_ and a C_18_ filtering pre-column. The analytical conditions were the following: liquid phase: methanol/water (90/10); flow rate: 0.5 mL/min; excitation wavelength: 337 nm; emission wavelength: 448 nm; retention time: 17, 23 and 42 min for So, Sa and C_20_ Sa, respectively.

### 4.6. Specific Serology for *Salmonella typhimurium*

#### 4.6.1. Qualitative Method

Seroconversion detection was obtained using a modified version of the method by Proux *et al*. [43]. Plates were sensibilised using the single LPS of *Salmonella* typhimurium (Sigma), and read in a spectrophotometer at 630 nm. Results were expressed as a proportion of the reference serum, with 0.4 as the positive threshold. 

#### 4.6.2. Quantitative Method

Quantitative serological analysis was performed on the sero-converted animals. An ELISA serum titration was performed on a range of serial ½ dilutions of the serums from an initial 1/100 dilution. OD was read at 630 nm. Comparisons were retained after confirmation of the linear proportionality of the OD value and the serum dilution.

### 4.7. Lymphocyte Proliferation Following Stimulation with a Mitogenic Agent or Specific Antigen

Fresh heparinised blood was diluted (1/15) in DMEM (Dulbecco’s Modified Eagle Medium; Eurobio, Les Ulis, France), seeded into 96-well plates (200 μL/well) and stimulated with a mitogenic agent (10 µg/mL concanavalin A, 50 ng/mL phorbol myristate acetate (PMA), 1 μg/mL ionomycin; Sigma, St. Quentin Fallavier, France) or specific antigen (10 μg/mL lipopolysaccharide from *Salmonella* spp; Sigma). The lymphocytes from control wells containing only blood remained unstimulated. After 48 h of incubation at 37 °C, 0.5 µCi of ^3^H-methyl-thymidine (ICN, Orsay, France) was added to each well. After another 24 h of incubation, cells were harvested through glass-fibre filters (Whatman, Maidstone, UK) by means of an automatic harvester (Titerteck-Skatron, Molecular Devices, St. Grégoire, France). Incorporation of thymidine was measured with a liquid scintillation counter (Kontron Instruments, St. Quentin en Yvelines, France), and the results were expressed according to the lymphocyte concentration in each animal [44].

### 4.8. Salmonella Detection and Count in Faeces and Tissues

*Salmonella* detection and a count were performed on the liver, spleen, diaphragm muscle, MLN, faeces and intestinal contents (jejunum, caecum, descending colon). The objective was to measure qualitative and quantitative *Salmonella* excretion (duration and intensity), as well as *Salmonella* translocation into the animal tissues. Unless otherwise stated, all media were purchased from AES France. For *Salmonella* detection, stomacher bags containing the 1/10 dilution in Buffered Peptone Water (BPW) samples were incubated at 37 °C for 16 to 20 h. One ml of the sample suspensions (pre-enrichment) was added to 10 mL Muller-Kauffmann Tetrathionate broth, and then incubated at 42 °C for 24 h. Medium was then isolated on an XLT4 selective agar plate, and incubated at 37 °C for 24 h. In addition, 100 µL of the sample suspensions was spot-inoculated on Modified Semisolid Rappaport-Vassiliadis Medium (MSRV) plates and incubated for 24 and 48 h at 41.5 °C. Characteristic migrations were streaked onto Rambach Agar plates (Merk, Molsheim, France). Any presumptive colonies of *Salmonella* were confirmed biochemically and serotyped. For counting, material (25 g) was diluted (1/10 *w*/*w*) in a BPW pre-enrichment medium, then mixed by stomaching for 30 s. The *Salmonella* count was performed using a most probable number (MPN) approach based on the miniaturisation of MSRV enrichment [45]. One mL of the diluted material was once again serially diluted briefly in TS tubes, in order to obtain concentrations of 10^1^ to 10^5^ CFU/mL. Dilutions were then replicated (triplicate), using a Spiral^®^ DS Plus plater (Interscience, St Nom-La-Breteche, France) on Petri dishes containing VBrif medium, then incubated at 37 °C for 24 h before counting. The MPN characteristic number was obtained by counting the number of positive wells in the dilutions using a system of three repetitions. The MPN characteristic number was converted into a number of *Salmonella* per gram of initial sample by using freeware developed by the “Institut Universitaire de Technologie” of Quimper (France) and based on De Man MPN Tables. The detection limit was 200 CFU/g of sample and the values were expressed as Log CFU/g. 

### 4.9. Aerobic Mesophilic Bacteria (AMB) Numeration in Faeces

Fifteen grams of thawed faeces were poured into buffered peptone water at 1:10 (*w*/*v*). The samples were serially diluted 10-fold and three dilutions of each sample were plated. Aerobic Mesophylic Bacteria (AMB) were counted using a Spiral^®^ DS Plus plater (Interscience, St Nom-La-Breteche, France) after 48 h of growth on Trypton Soja Agar plates (30 °C) as a global indicator for comparison of bacterial populations under different conditions. The concentration of bacteria in the original sample was expressed as Log CFU/g. 

### 4.10. Salmonella Immunohistochemical Analysis of the Peyer’s Patches

Immunolabelling of *Salmonella* was performed on serial 5 µm sections of the ileo-caecal junction in the vicinity of the Peyer’s patches. Sections were fixed overnight at 4 °C on glass slides in para-formaldehyde (4%). Then they were rinsed in a PBS bath, and subsequently a 1/50 anti-O_4,5_ rabbit serum diluted in the washing solution (PBS 1× + BSA 0.1%, Triton 0.5%) was placed on the slide. An isotypical negative control was performed for each series of sections (antibody anti-O_9_). Slides were placed in a moist environment for 40 min then rinsed three times for 5 min in the washing solution. A second anti-rabbit Ig antibody coupled to ALEXA 488 was placed on the slides which were then placed back into a moist environment for 40 min. A final washing (3 × 5 min) was performed before slide assembly and observation using an epi-fluorescent microscope (Olympus, Tokyo, Japan).

### 4.11. Capillary Single-Stranded Conformation Polymorphism (CE-SSCP) Analysis

In order to decrease inter-individual variability, the CE-SSCP analysis was performed on the four pooled DNAs obtained from the faecal samples of the four pigs in each pen. Characteristic faecal microbiota profiles for each pig group were obtained at the following dates: D − 6, D + 2, D + 7, D + 21 and D + 56.

#### 4.11.1. DNA Extraction/Amplification

DNA extractions from faecal samples were performed using a QIAamp DNA Stool Minikit (Qiagen, Hilden, Germany) [46]. One gram of thawed faeces was homogenised with 7 mL of lysis buffer, then 1.6 mL suspensions were used for DNA extraction. Extracted DNA was loaded onto 1% agarose gel in order to verify its quality. 

#### 4.11.2. PCR Amplification

For the total microbiota analysis, DNA was amplified from 1 μL of extracted DNA solution and added to the PCR mixture containing 1.3 μL of dNTP (Stratagene-Agilent Technologies, Santa Clara, CA, USA) (10 mM), 5 μL of buffer 10×, 1.3 μL of each primer (100 ng/μL), 0.5 μL of *pfu* turbo DNA polymerase (Stratagene-Agilent Technologies, Santa Clara, CA, USA) (2.5 U/μL), and 40.9 μL of high-purity water. The DNA sequence of the V3 region of DNA 16S was targeted for total bacteria recognition with already published primers [47] w49 (AGGTCCAGACTCCTACGGG) and w104* (*TTACCGCGGCTGCTGGCAC) (Sigma-Aldrich, Saint-Louis, MO, USA). Primer w104* was labelled with 5'-6 Fam fluorescent dye. Primer w49 was labelled with Hex fluorescent dye. These primers are specific to the Eubacteria phylogenic domain. The mix was run for 2 min at 94 °C, then 25 cycles of 30 s at 94 °C, 30 s at 61 °C, 30 s at 72 °C, and 10 min at 72 °C. PCR reactions were performed with a Gene Amp 9700 or 2400 (Applied Biosystems, Foster City, CA, USA). After amplification, 10 μL of the amplified product was run on a horizontal 2% agarose gel in TBE 1×, with a 100 bp DNA ladder in order to control PCR reactions (System Biosciences, Mountain View, CA, USA). Gels were stained with Ethidium Bromide (0.5 μg/mL) for 20 min and the images were captured under UV illumination by a video system.

#### 4.11.3. CE-SSCP Electrophoresis

Each PCR product was diluted in water according to the intensity of the signal observed on the agarose gel in order to obtain samples with standardized concentrations. One μL of the standardized proportion of the PCR product was diluted in highly purified water (1:5, *v*:*v*) and then mixed with 18.5 μL of sequencing buffer mix and 0.5 μL of internal standard HD 400 [rox] (Applied Biosystems, Foster City, CA, USA). After a 56 min denaturing step at 95 °C, the mix was quickly cooled on ice for 10 min before conducting capillary electrophoresis in a 3100-Avent Genetic Analyser (Applied Biosystems, Foster City, CA, USA). The CE-SSCP gel was composed of 6.22 g of CAP polymer (Applied Biosystem, Foster City, CA, USA), 1 g of glycerol (Invitrogen, Carlsbad, NM, USA), and 1 mL of 10× buffer (Applied Biosystem, Foster City, CA, USA), with Milli-Q water added to obtain 10 mL. Capillary Electrophoresis was run at 32 °C under 15 kV. Profiles were analyzed as described below.

### 4.12. Statistical Analysis

When possible, all data, except for the CE-SSCP Profiles (see below), were expressed as the mean ± Standard Error (SE) of results obtained from 12 animals up to D2 (data for the first sacrifice), and then for 8 animals, computed with SAS software (SAS, 2004). These data were analyzed by the ANOVA test for repeated and correlated series, after checking for homogeneity of the residual variance (Hartley’s test). When only two groups of data were compared, the *t*-test was used. Variable Differences were considered significant at *p* < 0.05 and trends were discussed at *p* < 0.1.

CE-SSCP Profile analyses were performed with GeneMapper™ (Applied Biosystems, Foster City, USA) and Bionumerics (Applied Maths, Sint-Martens-Latem, Belgium) software. The GeneMapper™ software was used to align the profiles obtained based on the internal migration standard. Similarity between profiles was studied by comparing the presence/absence of picks from profile to profile using the Bionumerics software. The Jaccard index [48] was calculated to establish inter-profile similarity using UPGMA method for dendrogram building.

## 5. Conclusions

The exposure of growing pigs to a moderate dietary concentration of fumonisins (11.8 ppm) was sufficient to induce a biological effect in pigs (see increase in Sa/So ratio). However, this intoxication did not increase the susceptibility of our SPF pigs to infection following *Salmonella* inoculation (5 × 10^4^ CFU). Despite *Salmonella*’s high invasiveness, an immuno-histochemical analysis of *Salmonella* did not reveal any translocation phenomena associated with exposure to fumonisins. However, our results suggest that moderate levels of fumonisins can induce inhibition of specific cellular response to *Salmonella*, although this effect remains to be confirmed. No effects on non-specific cellular response were observed.

Lastly, the inoculation of *Salmonella* alone did not affect faecal microbiota profiles. However, exposure to a moderate dietary concentration of fumonisins transiently affected the digestive microbiota balance. In cases of co-infection with fumonisins and *Salmonella*, the microbiota profiles were rapidly and clearly modified. Therefore under our experimental conditions and using SPF pigs, exposure to an average concentration of fumonisins affected the immune status of pigs and perturbed the digestive microbiota balance, with *Salmonella* exposure amplifying this phenomenon.
